# Guardians and Economy

**Published:** 1917-04-14

**Authors:** 


### FOOD IN WARTIME.*

### Guardians and Economy.

There was a general tendency when first the official food regulations were issued to take it for granted that they could not be intended to apply to institutions, either as regards inmates or staff, but were solely for home use. This misapprehension has been removed by the issue of a Local Government Board circular directing that after March 31 existing dietary tables which allow of an average consumption of flour, sugar, or meat in excess of the Food Controller's scale are to be revised after consultation with the medical officer. From now on the problem of living within the prescribed scale is a matter of vital import to the whole population, and incidentally the incompetence of the Guardians in certain localities to rule the households committed to their management in a spirit of common sense is being very glaringly brought into the open.

Some examples of the methods adopted in Poor-Law establishments may be quoted. The Liverpool Select Vestry have had a meeting to consider what can be done to reduce the diet table of the officers, and it has transpired that hitherto each officer has been entitled to draw to the amount of 11½ lbs. of meat and fourteen eggs a week. It is right to mention that the statement made by one of those present that nothing was ever returned to store after being issued was controverted by a lady Guardian, who stated that certain officers had either sent back a portion of the rations to which they were legally entitled or had refrained from drawing to the full amount so soon as the need for national economy became apparent. The blame should not in common fairness be laid at the door of the officers, who are not in any way responsible for the wasteful system. But the Guardians who inaugurated and maintained in the teeth of criticism after war began this scandalous extravagance ought to seek re-election in vain. If liberality to the staff be at the root of the business (which we cannot believe), by all means let salaries be raised all round, but even when plenty returns to the land there must be no harking back to obsolete ignorance and waste. The Liverpool estimates for officers' diet appear actually to be outdone in North Dublin, where, according to the *Irish Times*, the officers have hitherto been allowed every week, per head, bacon, 4 lbs.; mutton, 7 lbs.; butter, 2 lbs.; flour, 7 lbs.; potatoes, 14 lbs.; milk, 7 quarts.

The Birmingham Board of Guardians did not wait till the present crisis to bring their diet tables into order. At the outbreak of war the Board discontinued the customary and, in their opinion, wasteful system of issuing supplies in accordance with fixed dietary tables, and authorised the issue of food commodities in such quantities as the medical officers, masters, matrons, etc., were of opinion would satisfy the appetite of the various classes of inmates. The result was marked financial economy. Meantime, the officers of the various institutions under the Board voluntarily effected considerable economies. Thus the road was paved for the introduction

* Previous articles appeared on February 24, March 10, March 24, and March 31.

**FOOD IN WARTIME**—(*continued*).

of the war ration imposed by the Food Controller. An interesting report has been prepared showing the average quantities of bread, flour, meat, and sugar consumed per head per week by the officers at various Birmingham institutions during the six months ending September last:

*Average Consumption Per Head Per Week by Officers at various Birmingham Poor-Law Institutions during six months to September* 1916.

| | Bread lbs. | Flour lbs. | Meat lbs. | Sugar lbs. |
|---|---|---|---|---|
| Western Road ... | 5.5 | 0.87 | 5.25 | 0.62 |
| Erdington ... ... | 7.0 | 1.25 | 4.43 | 0.62 |
| Selly Oak ... ... | 5.1 | 0.75 | 5.60 | 0.46 |
| Monyhull Colony ... | 6.0 | 1.0 | 5.12 | 0.39 |
| Selly Oak Infirmary | 4.8 | 1.0 | 3.25 | 0.43 |

If the average amount of meat consumed per head per week without rationing be compared with the $11\frac{1}{2}$ lbs. allowed under the Liverpool scale, it will be seen how much the ratepayers have been plundered under the ration system. Even under the Food Controller's scale it will be seen that there will be loss instead of gain in sugar. The great difficulty in all these institutions is with bread and flour.

In the new scales for officers' diet drawn up in accordance with the Food Controller's directions, a large difference exists in regard to the sum set aside for sundries or extras. Formerly, this covered such things as pickles or fruit, and was not intended to purchase necessary articles of diet. But now that meat is so strictly limited in quantity it becomes necessary to allow for substitutes, and it is little use laying down what things shall be bought, for supplies vary in different localities, and people must eat what they can get. At Chichester the Guardians assign the sum of 4s. a week per head for additional articles in kind, over and above the following: Meat, bread, and sugar according to ration; flour, 1 lb.; tea, 8 oz.; butter, $\frac{1}{2}$ lb.; suet, $\frac{1}{2}$ lb.; bacon, $\frac{1}{2}$ lb.; milk, $3\frac{1}{2}$ pints; potatoes or other vegetables, $3\frac{1}{2}$ lbs. The total cost is said to work out at 18s. 6d. per week.

But at Mitford and Launditch institutions the Guardians have assigned each officer 1s. 6d. per week in addition to very much the same allowances. We are inclined to think 2s. 6d. a week is not too much to assign to unspecified articles of diet, considering present prices.

